# Supplementary material for: Patterns of Intron Gain and Loss in Fungi
Source: PLoS Biol. 2004 Nov 30;2(12):e422. doi: 10.1371/journal.pbio.0020422 (PMC532390; doi:10.1371/journal.pbio.0020422)
Supplement: Table S1 — Also available at http://genes.mit.edu/NielsenEtAl/. (4.3 MB ZIP). [file pbio.0020422.st001.zip › NielsenEtAl/html/359.html]

AN3841.1.NCU00896.1.MG01184.1.FG00688.1


```
 CLUSTAL W (1.82) Multiple Sequence Alignments - Introns Inserted


Sequence 1: NCU00896.1	704 aa
Sequence 2: MG01184.1	1101 aa
Sequence 3: FG00688.1	709 aa
Sequence 4: AN3841.1	706 aa
Alignment Length: 1117 aa
Number Identitical Residues: 311 aa
Alignment Score (without introns) 17184


MG01184.1 	------MSLPYRDINVHAAPDSYTFTSPSSPNAPALTIDRPTGDIRLTEGNALSGKRVQR
NCU00896.1	------MTLPFRDINVQAASDSYIFTSPSSPNAPALTIDRPTGDIRLSDASLLAGKRVAR
FG00688.1 	MAHLNTPKTPYRDINVKIANDSYTFTSPSSPDAPALVIDRPTGDVRLTDGSAASAKRTTR
AN3841.1  	---MVTSILPFRDINLHASPAHYAFTSPSSPDAPTLVVERPTGDLRLSNGSLSGAK---R
          	     :   *:****:: :   * *******:**:*.::*****:**::..  ..*   *

MG01184.1 	VSSIAGILGIISLRLD1KYVIIITKAQPAGRLKGHMVYRIVSTDLLPLRERQIRDAEEDR
NCU00896.1	VSSIAGILGVVRLRLD1KYIIVITKAQPVGRLRGHMVYKVVATEFLPLRERQIHDHDEDK
FG00688.1 	VSSIAGILGIIQLRLD1KYVIFITKAQPVGRLKGHMVYKVAATEIMPMRERLIHDPDEDV
AN3841.1  	ISSIAGILGIIKLKLD1KYIIVITKAQPMGRLRGQMIYKVAGTEFLPLRERPLHDHDEDA
          	:********:: *:** **:*.****** ***:*:*:*::..*:::*:*** ::* :** 

MG01184.1 	FLTLVRAFIKDSPMYFSYSVDLTNSFQRQSQHGNNTPLWMRADDRFFWNRFVQTDLINFR
NCU00896.1	FLNLLKGFIKSGPMYFSYSLDVTNTSQRQAQHDLSTPLWQRADDRFFWNRFLQSDLINFR
FG00688.1 	FIQLLKNFLASGPMYFSYSIDLTNSFQRQAQADISKPLWMQTDDRFFFNKYLQNDLIDFR
AN3841.1  	YLTMLKELLRTGPMYFSYTLDITNSFQRQSQSDMSLPMWKRADDRFFWNRFIQSDLIDFS
          	:: ::: ::  .******::*:**: ***:* . . *:* ::*****:*:::*.***:* 

MG01184.1 	Y---TGSRANPGAQKDVDPFILPVMFGMLEIHRTHFKNTPITLALITRRSRFRAGTRYLN
NCU00896.1	T---KGGRGSPAPQPGIDPYILPVIFGMFEIHPTTFKGTPITIALITRRSRHRAGTRYFS
FG00688.1 	T---RGSRSQSGAQPGVDPYILPCIFGMLEIKPTTFKGNPLTMVLITRRSRHRGGTRYFT
AN3841.1  	LGEHNTTSVRYGPQPGVDPYILPVIFGMLRITPAKVKSTTFTFALITRRSRHRAGTRYFS
          	 ..        ..* .:**:*** :***:.*  : .*...:*:.*******.*.****:.

MG01184.1 	RGLDAEGHAANYNETEQVLVMNDSGDGLGGFAGSSDMQSGKMGG--GKEMQIFSYVQTRG
NCU00896.1	RGIDAEGHASNYNETEQIVVLNDRGSGLGGFTGSGDMQSGKLGGSDGKEMQVLSYVQTRG
FG00688.1 	RGVDDEGHVANYNETEQVIILNDSNSGLGGYAGSSDMQSGKFGAGASQEMQIFSYVQTRG
AN3841.1  	RGIDEQGNVSNYNETEQIVILNDATGGLSGFAGGQTITKEKPAD-LGQDPQVMSFVQTRG
          	**:* :*:.:*******::::**  .**.*::*.  : . * .   .:: *::*:*****

MG01184.1 	SIPAFWAEVNALKYTPKLQVKSIEAALPAAAAHFREQVQIYGDNYLVNLVNQKGREKAAK
NCU00896.1	SVPVYWAEVNNLKYTPTIQLKSTEAAFPAAKAHFDEQIRLYGDNYLVNLVNQKGRETRVK
FG00688.1 	SVPTFWAEINSLRYVPKLEIRGIDTALTAAQKHFDEQIRLYGDNYLINLVNQKGREARVK
AN3841.1  	SVPVFWAEVNNLKYTPKLQVRGVETAVQAARKHFAEQIRLYGDNYLVNLVNQKGREERVK
          	*:*.:***:* *:*.*.::::. ::*. **  ** **:::******:*********  .*

MG01184.1 	EAYEEVVQRLSASSTKERTVADQRTDEKFHTIEP-TNAQNIFDRLHYIYFDFHAETKGNK
NCU00896.1	LAYEQMVDRLVSS-PKERVQSDALTDEKFHTIETSTKPQTSFDRLHYIYFDFHAETKGLQ
FG00688.1 	DSYEKMVEKLVSA-PRERQEADLLTAEKFTTIQP-EGKHQEFDRLHYIYFDYHSETKGMK
AN3841.1  	NAYEQLIRILVSS-PNELTEADDESSEKLHVLEP-DHPQKEMDRLHYVYFDFHNETKGLR
          	 :**:::  * :: ..*   :*  : **: .::.    :  :*****:***:* **** :

MG01184.1 	LHRAQLLLDQLKDVLEAQGYFCATESAASALDG-KMEVRTYQTSVVRSNCMDCLDRTNVV
NCU00896.1	MHRAQLLIDRMHEALIAQQYFRAVDSPASQVNGGRLEVRNLQTSVVRTNCMDCLDRTNVV
FG00688.1 	MHKAYALIEKLAEALEKQGYFRAVDTPS-NVDG-KLDARSYQTSVMRTNCMDCLDRTNVV
AN3841.1  	WHRAELLMDRLINGLNQGGYFRGLENPG--AAGGQLEARALQSSVVRTNCMDCLDRTNVV
          	 *:*  *:::: : *    ** . :...    *.:::.*  *:**:*:************

MG01184.1 	QSILGRHKLDRMFISLGLMAEGRTFKDEDPQFEFLFRNLWADNADMVSKSYAGTGAMKTD
NCU00896.1	QSMLARWTLDRMFIDLGLMARGSQFKDEDPAFEFMFRNMWADNADIVSNSYAGTGAMKTD
FG00688.1 	QSMFARHKLDRIFEDVGFMPRGSSFRDEDAAFEDMFRNLWADNADVVSNSYSGTGAMKTD
AN3841.1  	QSMLGRWALTRQLTEAGVLRPGEAAND-DQEFEDLFRNIWADNADVVSKAYSGTGALKTD
          	**::.*  * * : . *.:  *   .* *  ** :***:******:**::*:****:***

MG01184.1 	MTRMGIRTKAGALQDGNIAITRYFRNNFLDGPRQDSFDIFLGAYQPAAAANIGSGLIFAD
NCU00896.1	LTRTGKRTKAGALADANIAVTRYCKNNFFDGPRQDAFDLFLGVYQPG-AANIGSQLVFVD
FG00688.1 	VTRTGNRTKVGALQDARIGVTRYFRNNFFDGPRQDSFDLFLGTYRPG-SANIGTTLVFTD
AN3841.1  	FTRTGQRTRAGMVQDLSNSITRYVRNNFLDGPRQDGFDVFLGAYLPP-ESTLGNLRIFVD
          	.** * **:.* : *   .:*** :***:******.**:***.* *   :.:*.  :*.*

MG01184.1 	RRPLLIQAIPYIMGFSSFLVFVALFTKRMPDAAVMPLRLFTLFFFAVSAWCAYFIINNGI
NCU00896.1	RRPVLIQAIPYLAAFSLFFVFVGMWTPRLPDSAVWPMRLFIIFWTAVAAWSLTFIISNGM
FG00688.1 	RRPILIQSIPYILAFSVFIILTGLFTRRAPDASALPLRIFLFFWMAIAAWSFYFIWNHGM
AN3841.1  	RRPLIIQSIPYIFAAGLFMIIIALFTRRLPDAAVWPLRLFVVFWLLISGWCARFMLAHGM
          	***::**:***: . . *::: .::* * **::. *:*:* .*:  ::.*.  *:  :*:

MG01184.1 	LY0VNWPKLNPRPWAQEGYQDTISKIRR-DKIIGPFVA--KHERGLSTARYISAEEG~KK
NCU00896.1	LY0VNWPKLVPRPWAVEGYHETITKVVRKDKILGPLVA--KHERGLSTARYINAEEG1LG
FG00688.1 	LY0VNWPRLNPRPFAVEGYNEHFAKARK-DSVIGTYVA--KHERGLSTARYLNAEEG~KK
AN3841.1  	LY0VNWPKLNTPAAGSEGYQDALIKARS-DPVIGQLLPSRRHQRGYSNARLGFLEEG~KT
          	** ****:* . . . ***:: : *    * ::*  :.: :*:** *.**    ***   

MG01184.1 	RVE2WHKHQQLYHFFCAFRMCVFLLWDGNAAAW2LAYLILLSYSCKVTLASPVSGRPRCV
NCU00896.1	---~-----------------------------~--------------------------
FG00688.1 	RIE~-----------------------------~--------------------------
AN3841.1  	RIE~-----------------------------~--------------------------
          	  .                                                         

MG01184.1 	SPTVSQPTPIASGLVILGYHDGDIGRPLQVPYGKVVVTDSDRMMWGYCGEVSVGDETCRL
NCU00896.1	------------------------------------------------------------
FG00688.1 	------------------------------------------------------------
AN3841.1  	------------------------------------------------------------
          	                                                            

MG01184.1 	AGACVDSQGCSDGCAVGATPKTRLSTSKDGAETVLLKC2SSESHSSICTTATWTRRDGAA
NCU00896.1	--------------------------------------~---------------------
FG00688.1 	--------------------------------------~---------------------
AN3841.1  	--------------------------------------~---------------------
          	                                                            

MG01184.1 	FASLGCGPTATTAHILEEPLYRVFIGQETLFPSGETARAGRSESGLDAVTVAVPVEIGYE
NCU00896.1	------------------------------------------------------------
FG00688.1 	------------------------------------------------------------
AN3841.1  	------------------------------------------------------------
          	                                                            

MG01184.1 	AAQATETGTPESGVGSHDSVE1TSATTKILETSQTDYPTPPLPPPPPPAATPTPRASLTG
NCU00896.1	---------------------~--------------------------------------
FG00688.1 	---------------------~--------------------------------------
AN3841.1  	---------------------~--------------------------------------
          	                                                            

MG01184.1 	SHPISNQPLQSLTRSVSLVTEVPATATTSAAAESPQVGGLAPGQVGGIVVGSIAGAALLA
NCU00896.1	------------------------------------------------------------
FG00688.1 	------------------------------------------------------------
AN3841.1  	------------------------------------------------------------
          	                                                            

MG01184.1 	FGTLYIAWRHLLSRFSMAMGSIYMTLYHPRPAPASTSQAHAAGP
NCU00896.1	--------------------------------------------
FG00688.1 	--------------------------------------------
AN3841.1  	--------------------------------------------
          	
```
